# Role of Protein Carbonylation in Skeletal Muscle Mass Loss Associated with Chronic Conditions

**DOI:** 10.3390/proteomes4020018

**Published:** 2016-05-06

**Authors:** Esther Barreiro

**Affiliations:** 1 Pulmonology Department and Research Group on Muscle Wasting and Cachexia in Chronic Respiratory Diseases and Lung Cancer, Institut Hospital del Mar d’Investigacions Mèdiques (IMIM)-Hospital del Mar, Health and Experimental Sciences Department (CEXS), Pompeu Fabra University (UPF), Barcelona Biomedical Research Park (PRBB), C/Dr. Aiguader, 88, Barcelona E-08003, Spain; ebarreiro@imim.es; Tel.: +34-933-160-385; 2 Centro de Investigación en Red de Enfermedades Respiratorias (CIBERES), Instituto de Salud Carlos III (ISCIII), Barcelona E-08003, Spain

**Keywords:** oxidants, protein carbonylation, skeletal muscle wasting and dysfunction, disuse muscle atrophy, aging, cigarette smoking, COPD, cancer-induced cachexia, septic muscles

## Abstract

Muscle dysfunction, characterized by a reductive remodeling of muscle fibers, is a common systemic manifestation in highly prevalent conditions such as chronic heart failure (CHF), chronic obstructive pulmonary disease (COPD), cancer cachexia, and critically ill patients. Skeletal muscle dysfunction and impaired muscle mass may predict morbidity and mortality in patients with chronic diseases, regardless of the underlying condition. High levels of oxidants may alter function and structure of key cellular molecules such as proteins, DNA, and lipids, leading to cellular injury and death. Protein oxidation including protein carbonylation was demonstrated to modify enzyme activity and DNA binding of transcription factors, while also rendering proteins more prone to proteolytic degradation. Given the relevance of protein oxidation in the pathophysiology of many chronic conditions and their comorbidities, the current review focuses on the analysis of different studies in which the biological and clinical significance of the modifications induced by reactive carbonyls on proteins have been explored so far in skeletal muscles of patients and animal models of chronic conditions such as COPD, disuse muscle atrophy, cancer cachexia, sepsis, and physiological aging. Future research will elucidate the specific impact and sites of reactive carbonyls on muscle protein content and function in human conditions.

## 1. Introduction

Muscle dysfunction, in which muscle fibers undergo a reductive remodeling, is a common systemic manifestation in highly prevalent conditions such as chronic heart failure (CHF), chronic obstructive pulmonary disease (COPD), cancer cachexia, and critically ill patients. Muscle mass loss and dysfunction may also aggravate the number of exacerbations in chronic respiratory patients, thus further impairing the patients’ quality of life and physical activity and/or exercise performance [1,2,3,4,5]. In addition, skeletal muscle dysfunction and impaired muscle mass may predict morbidity and mortality in patients with chronic cardiac and respiratory diseases, independently of the severity of their lung disease [6,7,8,9,10,11,12,13]. Moreover, loss of muscle function and mass is also common in the elderly, usually known as sarcopenia, which may further impair disease progression and prognosis. Therefore, maintenance of an adequate muscle mass and performance is indispensable for patients with chronic diseases and systemic manifestations to maintain their daily life activities and improve survival. Moreover, a complete evaluation of these patients should include the assessment of their nutritional status, muscle mass and performance and specific pharmacological and non-pharmacological therapeutic strategies should aim at restoring the overall performance and functional capacity of those patients.

**Figure 1:**
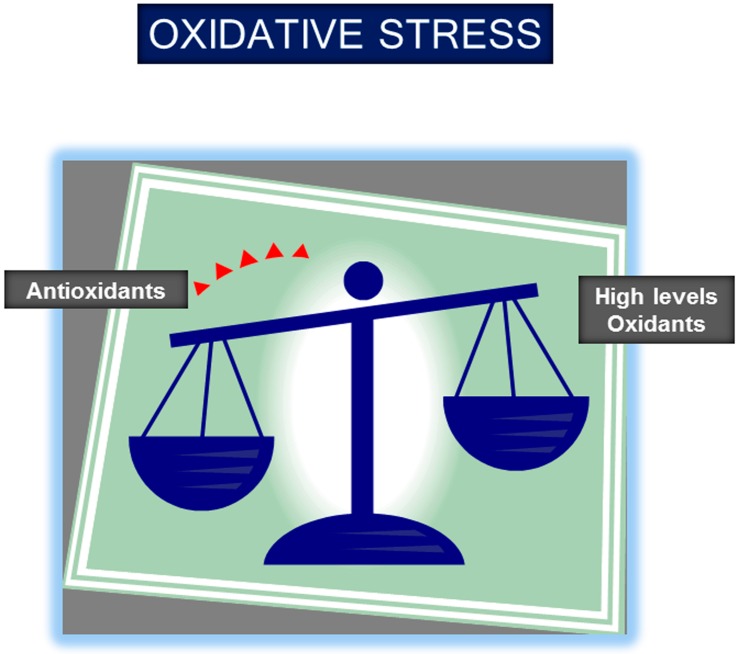
Oxidative stress results from the imbalance between the production of oxidants and the effects of antioxidants in favor of the former.

**Figure 2:**
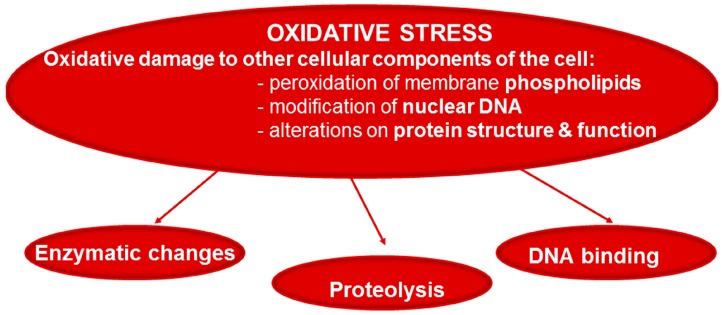
Reactive oxygen species (ROS) that are not scavenged by cellular antioxidants oxidize key cellular structures such as membrane lipids, nuclear DNA, and proteins. Oxidative damage of proteins exerts different effects such as alteration of enzyme activity and DNA binding of transcription factors and may also render the proteins more susceptible to be degraded.

Although the etiology of muscle mass loss and dysfunction in chronic conditions is still under investigation, several common factors such as oxidative stress, inflammation, hypoxia, abnormal nutritional status, and deconditioning (disuse) have already been suggested. Oxidative stress, defined as the imbalance between oxidants and antioxidants in favor of the former (Figure 1), was shown to be a key contributing factor to the respiratory and limb muscle dysfunction of patients with COPD [14,15,16,17,18,19,20,21,22,23,24,25,26,27,28,29,30], muscles of animals with experimental cancer cachexia [31,32,33], and sepsis [34,35,36,37,38,39,40], and in elderly subjects [41,42,43]. High levels of oxidants may alter the function and structure of key cellular molecules such as proteins, DNA, and lipids, eventually leading to cellular injury and cell death (Figure 2) Moreover, protein oxidation including protein carbonylation was demonstrated to modify enzyme activity and DNA binding of transcription factors, while also rendering proteins more prone to proteolytic degradation (Figure 2) [44,45,46,47]. Given the relevance of protein oxidation in the pathophysiology of many chronic conditions and their comorbidities, the current review focuses on the analysis of the different studies in which the biological and functional significance of the modifications induced by reactive carbonyls on proteins have been explored so far in skeletal muscles of patients and animal models of chronic conditions such as COPD, disuse muscle atrophy, cancer cachexia, sepsis, and physiological aging.

## 2. Production of Oxidants and *in Vivo* Protein Carbonylation

Oxygen free radicals are produced *in vivo* in large quantities under a variety of conditions such as exposure to high oxygen tensions, during ischemia-reperfusion, and inflammatory conditions [48,49,50]. Reactive oxygen species (ROS) are redox derivatives of molecular oxygen, in which the parent molecule is the superoxide anion. Other ROS include hydroxyl radicals (OH^•^), hydroperoxyl radicals (HOO^•^), and hydrogen peroxide (H_2_O_2_), which is not a free radical as it has an even number of electrons. Current evidence shows that ROS may also play a relevant role in the regulation of signaling pathways involved in muscle adaptation to exercise and inactivity. The magnitude and time-course production of ROS determine their effects on tissues. As such, a moderate rise of oxidants during a short period of time activates signaling pathways that lead to cell adaptation and protection against further stress within the myofibers [51,52]. Nevertheless, high levels of ROS synthesized over long periods of time may lead to the activation of signaling pathways that accelerate proteolysis and eventually cell death [52].

Metal ion-catalyzed reactions of the Fenton and Haber-Weiss types are counted among the most significant mechanisms of protein oxidation in *in vivo* systems. Thus, carbonyl derivatives (aldehydes and ketones) are formed by reaction of oxidants with lysine, arginine, proline, and threonine residues of the protein side-chains. Moreover, direct reactions of proteins with ROS may also lead to the formation of protein derivatives or peptide fragments containing highly reactive carbonyls. In addition, secondary reactions of primary amino groups of lysine residues with reducing sugars or their oxidation products (glycation and/or glycoxidation reactions) may also generate reactive carbonyls in proteins [53,54].

Another mechanism of reactive carbonyl group formation is by Michael-addition reactions of lysine, cysteine, or histidine residues with α,β-unsaturated aldehydes generated during the peroxidation of polyunsaturated fatty acids of the membranes [54,55,56]. For instance, 4-hydroxy-2-nonenal (HNE) reacts with lysine, cysteine, and histidine residues of proteins to form Michael adducts that can be stabilized and further detected using a selective antibody [55]. Malondialdehyde (MDA) reacts with lysine residues to form Schiff base adducts that can also be detected in tissues using selective antibodies [56]. Other forms of protein oxidation include the oxidation of aromatic amino acid residues, cyclic oxidation and reduction of methionine, protein-protein cross linkage, chlorination reactions, oxidation of free amino acids, and modifications of proteins induced by reactive nitrogen species (RNS).

Oxidative stress-induced cellular damage in tissues can be identified using different methodologies. For instance, carbonyl-modified proteins are commonly detected using hydrazide, which reacts specifically with protein carbonyls in the form of aldehydes via Schiff base formation, and are stabilized with sodium cyanoborohydride [57]. Biotin hydrazide is commonly used to label and identify carbonylated proteins. In several types of samples such as yeast whole-cell lysates and in liver homogenates, enriched, biotinylated proteins have been consistently identified using a gel-free approach and avidin-affinity chromatography coupled with direct analysis by mass spectrometry [58,59]. Moreover, carbonylated proteins from enriched skeletal muscle mitochondria were also identified using a stable isotope labeling approach (isobaric tags for relative and absolute quantitation, iTRAQ) [57]. These methodologies are useful to overcome the background problems related to binding of avidin to non-carbonylated proteins (endogenous biotinylated proteins and hydrophobic and electrostatic interactions of avidin) when detecting protein carbonyls in gel-free systems [57].

**Figure 3:**
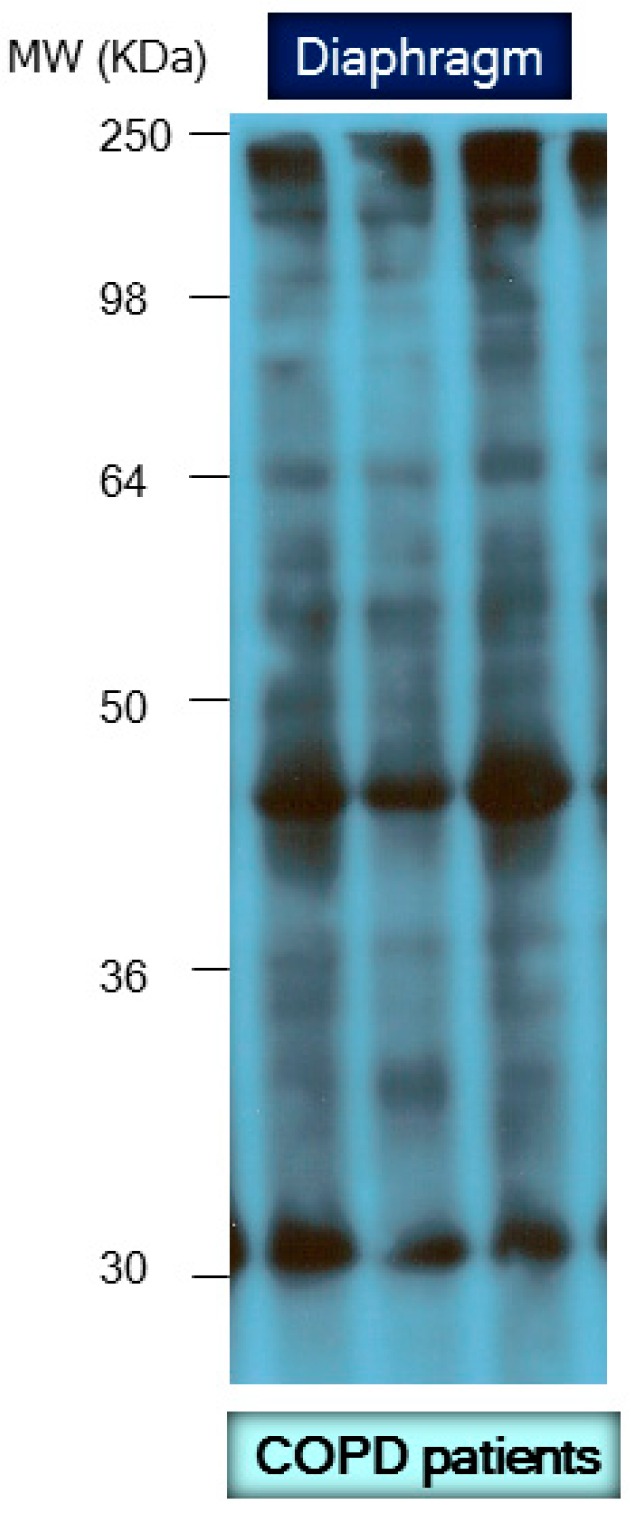
Representative 1D immunoblot with molecular weights corresponding to the detection of carbonylated proteins in crude muscle homogenates of the diaphragm muscle in three patients with severe chronic obstructive pulmonary disease (COPD). For detailed information see reference [23].

**Figure 4:**
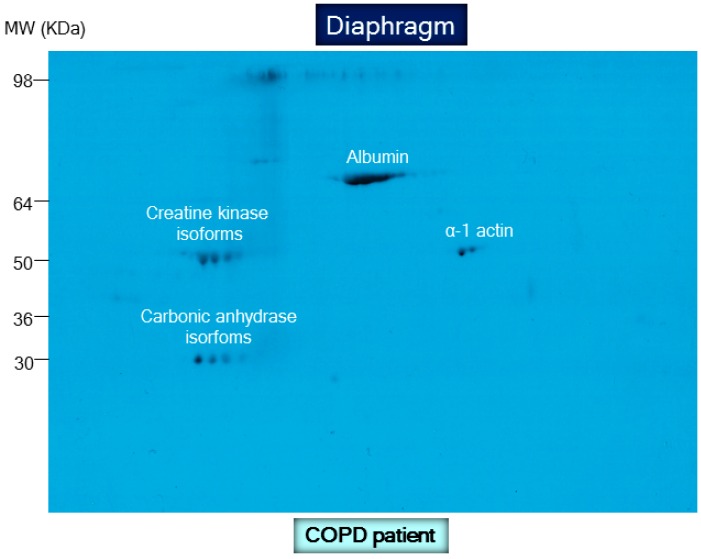
Representative 2D immunoblot with molecular weights corresponding to the detection of carbonylated proteins in crude muscle homogenates of the diaphragm muscle in one patient with COPD. For detailed information see reference [23].

**Figure 5:**
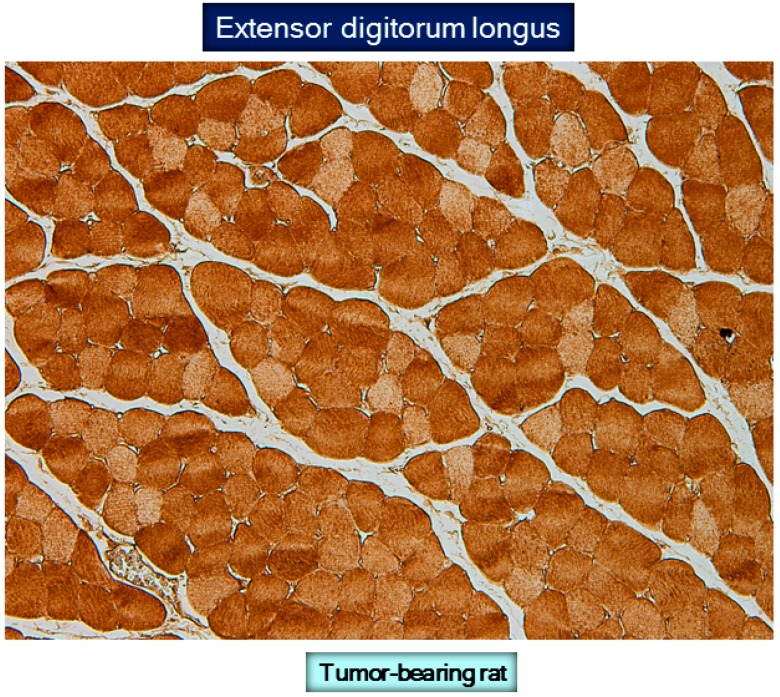
Representative example of reactive carbonyl immunostaining in the extensor digitorum longus muscle of a tumor-bearing rat. Note that the intensity of the protein carbonylation staining differed among the fibers. A darker intensity was observed in the fast-twitch fibers compared to the slow-twitch fibers. For more information see reference [33].

Importantly, the carbonyl assay also appears to be an accurate, reliable method to quantify protein oxidation in a simple and robust manner [60]. This assay enables the detection of reactive carbonyl groups after their chemical reaction with 2,4-dinitrophenylhydrazine (DNPH) to form 2,4-dinitrophenylhydrazone (DNP) moieties using several techniques such as enzyme-linked immunosorbent assay (ELISA), immunoblotting including 1D and 2D electrophoresis (Figure 3 and Figure 4, respectively), and immunohistochemistry (Figure 5). Despite the fact that oxidative modifications other than protein carbonylation may also occur in tissue or cell proteins, the reliability, simplicity, and convenience of the carbonyl assay make it extremely useful and meaningful as an index of total protein oxidation in biological systems. Differences in the protein carbonyl content among several samples reflect their resistance or susceptibility to oxidative stress. In this regard, different studies from several groups including our own have demonstrated increases in the levels of protein carbonylation as well as identification using proteomics analyses in tissues under different conditions [14,15,16,17,18,19,23,30,31,32,34,35,36,40,41,61,62,63,64,65,66,67,68]. Indeed, protein oxidation has been shown to play a crucial role in the pathophysiology of multiple disorders such as degenerative diseases, aging, atherosclerosis, cancer, and skeletal muscle dysfunction associated with acute and chronic conditions [14,41,69,70]. The most relevant results of those investigations are discussed below.

## 3. Oxidants and Skeletal Muscle Contraction

Oxidants derive from two parent molecules: O_2_^−^ and NO within the myofibers. Although ROS are needed for normal cell adaptation to environmental stimuli [71,72,73,74] they may also overwhelm tissue antioxidant capacity when produced at high levels in inflammatory-immune conditions. Specifically, in skeletal muscle fibers, ROS are normally synthesized at low levels and are absolutely required for normal force production [71,72,73,74]. However, when levels of ROS are either reduced by the action of free radical scavengers or excessively produced under inflammatory conditions muscle force generation may be considerably impaired, leading to further muscle dysfunction and fatigue [71,72,73,74]. In resting and contracting skeletal muscle fibers, superoxide anion and NO are the primary free radicals being generated. As abovementioned superoxide anion gives rise to hydrogen peroxide, hydroxyl radicals, and other oxidants that form the reactive ROS cascade. NO targets sulfhydryl groups in various proteins through the process of *S*-nitrosylation but can also react with superoxide anion to form highly reactive nitrogen species (RNS) such as peroxynitrite (ONOO^−^) and nitrogen dioxide (NO_2_).

In resting muscles, ROS and RNS are generated at low levels, and they promote physiological functions including regulation of the contractile process, glucose uptake, and blood flow. During strong contractions or under pathophysiological conditions [75], ROS and RNS are synthesized at higher rates, which may overcome tissue antioxidant capacity, thereby leading to the development of oxidative stress (Figure 1). Oxidants including peroxynitrite target several structures within the muscle fibers such as contractile proteins, key metabolic enzymes, sarcoplasmic reticulum, and ryanodine receptors.

In skeletal muscles, ROS are mainly produced by the mitochondrial respiratory chain, especially during contractile activity. ROS can also be derived from other sources such as nicotinamide adenine dinucleotide phosphate hydrogen (NADPH) oxidase [76], xanthine oxidase, microsomal system p-450, arachidonic acid, and chemical reactions with transition metals [71,77,78]. NO·is continuously produced by nitric oxide synthases (NOS) in skeletal muscle fibers, and its generation is enhanced during contraction [79]. Three isoforms of NOS have been identified so far: constitutive endothelial (eNOS) and neuronal (nNOS), which are calcium-dependent, and inducible (iNOS), which is calcium-independent. Several RNS are formed inside skeletal muscle fibers, including the highly reactive peroxynitrite, formed by reaction of NO·with O_2_^−^, which triggers post-translational modifications of proteins including nitration of tyrosine residues leading to the formation of nitrotyrosine, which is a major biological marker of nitrosative stress. On the other hand, peroxynitrite also exerts direct oxidative effects (oxidative stress) on several molecules and structures within the muscle fibers.

## 4. Antioxidants in Skeletal Muscle Fibers

Skeletal muscle fibers possess strong antioxidant systems that protect the myocytes from potential deleterious effects of ROS. For instance, the antioxidants CuZn-superoxide dismutase (SOD1), catalase, and glutathione peroxidases are present in the sarcoplasm, while Mn-SOD (SOD2) and glutathione peroxidase-1 and -4 are localized within the mitochondrial matrix. Other thiol-based antioxidant proteins such as thioredoxins and peroxiredoxins are also abundantly expressed inside the myocytes. In addition, non-enzymatic antioxidant systems complement the action of the antioxidant enzymes such as the lipid soluble compounds vitamin E, carotenes, and ubiquinol, which are localized to cell membranes. For instance, ascorbic acid, urate, lipoate, and glutathione, the most abundant non-protein thiol, are water soluble and widely distributed within the muscle fibers. The ratio of reduced to oxidized glutathione (GSH/GSSG) is an indicator of the redox tissue potential. In fact, thiol oxidation is also considered to be a sensitive marker of oxidative stress that has been clearly implicated in muscle fatigue [80].

## 5. Protein Carbonylation in Disuse Muscle Atrophy

Significant loss of muscle mass and function are seen during prolonged periods of muscle disuse as a result of immobilization, physical inactivity, diaphragm unloading via mechanical ventilation, chronic bed rest, or spaceflight. Identification of the biological mechanisms involved in disuse muscle atrophy is of paramount importance to develop therapeutic strategies that can prevent this form of muscle wasting and recover function. Furthermore, muscle atrophy may also be associated with acute and chronic conditions such as COPD, sepsis, cancer, aging, and other diseases. Animal models of immobilization have shown that loss of muscle mass and performance is the result of a decrease in muscle protein anabolism and an increase in the rate of proteolysis [77,81].

In the last decades, seminal studies showed that oxidative injury also takes place during periods of disuse in skeletal muscles of animals exposed to hindlimb suspension [82,83,84,85] and in the unloaded diaphragm during prolonged mechanical ventilation in rodents [86,87] and humans [88]. Despite the fact that identification of sources of ROS production within the myofibers during prolonged inactivity remains a matter of investigation, several pioneering studies have already shown that xanthine oxidase and mitochondria are the main contributors to oxidant production in mechanical ventilation-induced diaphragm atrophy [89,90]. Furthermore, levels of several markers of oxidative stress including protein carbonylation were significantly increased, while antioxidant levels were decreased in several organs and heart of rodents exposed to an immobilization protocol of the four limbs for two weeks [91,92].

Oxidative stress may contribute to disuse muscle atrophy by enhancing protein catabolism via activation of several proteolytic systems: calpains, caspase-3, and the ubiquitin-proteasome system in skeletal muscles [52]. Disturbances in calcium homeostasis by ROS may lead to cellular calcium overload, through several mechanisms, which may activate calpains [52,77]. In different cell types, increased levels of ROS may also activate caspase-3 [93], which degrades intact actin-myosin complexes. Furthermore, oxidative stress was also shown to promote proteolysis via the ubiquitin-proteasome system in skeletal muscle myotubes [94]. In conclusion, on the basis of recent evidence, increased ROS production seems to participate in the pathophysiology of disuse muscle atrophy. However, future studies are needed to specifically identify the molecular sources of ROS synthesis and their target cellular structures within the myofibers in different models of disuse.

## 6. Protein Carbonylation in Aging Muscles

In skeletal muscles, aging is defined as a decline in performance and fitness with advancing age. Even in healthy individuals, muscles become weaker and less powerful as age progresses, impairing their ability to perform essential physical activities of daily life, while increasing prevalence for falls and morbidity. As a matter of fact, the deleterious effects of aging are best observed in postmitotic tissues such as skeletal muscles and neurons, where damaged or lost cells cannot be replaced by the mitosis of intact ones. The overall loss of muscle mass, quality, and strength is generally known as sarcopenia, which includes among various features the atrophy of fast-twitch muscle fibers, a decrease in mitochondrial volume and enzyme content, mutations in mitochondrial DNA, and reduced muscle respiratory rates [95,96,97].

Studies in humans. Other studies have focused their attention on the assessment of the implications of oxidative stress in muscles obtained from elderly humans. For instance, increased levels of oxidized glutathione, lipid peroxidation, both Mn-SOD and catalase activity, protein carbonylation, and DNA oxidation were shown in the quadriceps muscles of elderly patients undergoing orthopedic surgery [42,43,98]. Moreover, Mn-SOD activity was also shown to be higher in the rectus abdominis of elderly patients undergoing abdominal surgery [99], while the activity of catalase and glutathione transferase was reduced in satellite cells of limb muscles in elderly subjects [100].

In another investigation [41], in which muscles from healthy senescent individuals were compared to muscles of young subjects, total reactive carbonyls, MDA-protein adducts, and protein tyrosine nitration were shown to be greater in their respiratory muscles (external intercostal), while only MDA-protein adducts and tyrosine nitration were higher in their limb muscles. In the same study [41], a *post hoc* analysis specifically revealed that elderly women exhibited greater levels of both total protein carbonylation and nitration, as well as of Mn-SOD content in the intercostal muscles, while the elderly men only showed an increase in MDA-protein adducts in their limb muscles. Importantly, the increased ROS-mediated effects on muscle proteins observed in the external intercostals of healthy elderly humans may have additional deleterious effects under pathological conditions such as immobilization, mechanical ventilation, and COPD [41]. Furthermore, these data [41] also suggest the existence of a sex-related regulation of redox balance in skeletal muscles of elderly subjects, which may eventually offer a target for therapeutic intervention in the aging process. Future investigations should focus their attention on the identification of the oxidatively modified proteins in senescent muscles and whether they could eventually trigger loss of function and enhanced protein degradation, thus having a negative impact on muscle mass and performance in elderly subjects.

Studies in animals. Several factors have been implicated in the etiology of sarcopenia such as the loss of growth hormone, a reduction in estrogen and androgen production [101], impaired glucose and/or fatty acid metabolism, nitrogen imbalance, decreased muscle protein synthesis, reduced physical activity, and oxidative stress [100,102,103]. In fact, aging has also been postulated to be the consequence of a greater accumulation of ROS-mediated deleterious effects on tissues than those normally neutralized by intracellular antioxidant defenses [104]. It is nowadays commonly accepted that oxidative stress is primarily involved in the etiology of aging, especially in those tissues with high levels of oxygen metabolism such as skeletal muscles [103,104]. As such, the diaphragm of senescent rodents was shown to increase its oxidative capacity [105], as well as its content in antioxidant enzymes [106]. Furthermore, an age-dependent loss in sarcoplasmic reticulum Ca^2+^-ATPase isoform 2a activity was also shown as a result of 3-nitrotyrosine accumulation in limb muscles of senescent rats [107]. Moreover, limb muscles from senescent mice (18 months old) also exhibited a reduction in the function of the mitochondrial respiratory chain, which was associated with increased levels of protein carbonylation [108].

Importantly, Feng *et al.* [109] expertly showed using iTRAQ-based quantitative proteomics that fast-twitch muscles (semimembranosus, plantaris, extensor digitorum longus, and tibialis anterior) had twice as many carbonylated proteins in the mitochondria than was observed in the slow-twitch muscles (soleus) of the same rats in an age-dependent manner (12- and 26-month old animals). Besides, in the study [109], increases in carbonylation state were detected in 22 proteins according to the age of the rats. Interestingly, the proteins identified to experience those changes were part of cellular pathways involved in fatty acid and glucose metabolism, reported to be impaired in senescent muscles [109].

## 7. Protein Carbonylation in Muscles Exposed to Chronic Cigarette Smoke

Studies in humans. Protein oxidation, as measured by either reactive carbonyls or MDA-protein adducts, was significantly increased in the muscles of healthy smokers, who had not developed any respiratory or cardiovascular disease at the time of study entry. Glycolytic enzymes, creatine kinase, carbonic anydrase-3, ATP-synthase, and structural proteins were shown to be more carbonylated in the quadriceps of the smokers and patients with severe COPD. Importantly, chronic exposure to cigarette smoke induced no significant rise in muscle inflammation in either healthy smokers or rodents [17]. The function of the quadriceps muscle was also significantly reduced in the healthy smokers than in control subjects [17]. It would be possible to conclude from these findings that protein carbonylation may have partly contributed to such a decrease in healthy smokers. The mechanisms whereby increased carbonylation of muscle proteins impair muscle contractile performance remain to be further elucidated.

Studies in animals. Respiratory and limb muscles of guinea pigs chronically exposed to cigarette smoke also exhibited an increase in protein carbonylation levels compared to non-exposed control animals as early as 3 months of exposure [17]. Glycolytic enzymes, creatine kinase, carbonic anydrase-3, ATP-synthase, and structural proteins were also shown to be more carbonlyated in the muscles of the rodents chronically exposed to cigarette smoke than in the control guinea pigs [17]. Besides, the proportions of slow-twitch muscle fibers were moderately decreased only in the diaphragms of the cigarette smoke -exposed rodents at 6 months. Importantly, animals in this model did not develop emphysema and only signs of bronchiolar abnormalities were seen in the airways of the study groups late in the course of cigarette smoke exposure [17]. Significant inflammatory events did not develop in any of the analyzed muscles [17].

In another investigation [64], we also demonstrated that highly abundant proteins involved in glycolysis, energy production and distribution, carbon dioxide hydration, and muscle contraction were shown to be strongly carbonylated in respiratory and limb muscles of AKR/J mice chronically exposed to cigarette smoke. In this investigation, mice developed clear signs of emphysema at 6 months of study [64]. In fact, the strain of AKR/J mice was shown to be extremely susceptible to the development of lung emphysema as opposed to other animal models of chronic cigarette smoke exposure [110]. Another relevant finding in the model of AKR/J mice chronically exposed to cigarette smoke [64] was the association encountered between the degree of emphysema and levels of protein carbonylation detected in the diaphragms of the exposed rodents. Such an association prompted us to conclude that the mechanical loads imposed by the lungs onto the diaphragm may also account for the increased oxidation of its muscle proteins, as was shown to occur in severe COPD patients [14,23].

Importantly, in both animal models [17,64], the effects of oxidants on muscle proteins were observed simultaneously in both respiratory and limb muscles, suggesting that chronic cigarette smoke exposure probably exerted direct deleterious effects on all muscles of the exposed animals. Furthermore, the effects of oxidants on the rodent muscles occurred at an earlier stage than the effects observed in the respiratory system [17]. These findings reinforce the concept that cigarette smoke *per se* is likely to be involved in direct tissue toxicity (aldehydes, peroxides, nitrogen oxides, and peroxyl radicals, among others) in skeletal muscles of the exposed animals, regardless of the structural alterations found in the respiratory system. Interestingly, similar findings were also reported in previous investigations [111,112,113,114,115,116,117], in which a rise in different oxidative stress markers was also demonstrated in the blood, lungs, and other organs of human smokers and animals chronically exposed to cigarette smoke. Besides, direct oxidation of other proteins such as albumin has also been shown in response to direct exposure to cigarette smoke in *in vitro* models [118,119].

Another relevant finding in both animal models of chronic cigarette smoke exposure [17,64] was the reduction in total body weight gain observed in guinea pigs [17] and mice [64] chronically exposed to cigarette smoke. Interestingly, changes in body weight gain took place at an earlier stage than the development of the respiratory abnormalities [17,64]. The exact mechanisms whereby decreased body weight gain occurs in animals chronically exposed to cigarette smoke and whether they share similarities to those involved in muscle mass loss and dysfunction in smokers and COPD patients remain unanswered. Enhanced muscle protein carbonylation could be a relevant contributing trigger. However, whether decreased food intake and/or physical activity may play a significant role in the reduced body weight gain of animals chronically exposed to cigarette smoke also needs to be confirmed and further elucidated. Besides, identification of the chronological sequence of events involving protein carbonylation, muscle mass and total body weight loss, and the development of the respiratory disease in cigarette smoke exposure also deserves attention.

## 8. Protein Carbonylation in Skeletal Muscle Dysfunction and Mass Loss in COPD

### 8.1. Muscle Dysfunction and Mass Loss in COPD

COPD is a highly prevalent condition that imposes a significant economic burden worldwide as a consequence of acute exacerbations and comorbidities. In COPD patients, skeletal muscle dysfunction is a common systemic manifestation that affects both respiratory and limb muscles [120], resulting in a significant impairment of their quality of life. Quadriceps muscle dysfunction appears in one third of patients, even at very early stages of the disease when severe airway obstruction has not yet developed [7]. Additionally, quadriceps weakness and reduced muscle mass as measured by mid-thigh cross-sectional area were also shown to be good predictors of COPD mortality [6,7,8,9]. Skeletal muscle dysfunction in COPD patients is characterized by reduced muscle strength and endurance, probably due to the interaction of different systemic and local factors.

Skeletal muscle dysfunction in COPD is also highly dependent on the specific function of the muscle [121]. In patients with severe COPD, the mechanical loads imposed by the respiratory system, which modify the resting length of the diaphragm, play a major role in their respiratory muscle dysfunction. Moreover, the diaphragm must remain active throughout the existence of the individual. Interestingly, biological and structural factors are also involved in the reduced contractile performance observed in the patients [68,121]. Peripheral muscles, which do not have to contract at a specific respiratory length, are negatively affected by several biological and structural factors in severe COPD patients [19].

In general, lower limb muscles are more adversely affected than inspiratory muscles, probably as a result of disuse or deconditioning [122]. For instance, the vastus lateralis muscle of patients with severe COPD consistently exhibits a slow-to-fast fiber type switch [18,19,123]. Nonetheless, in the same patients, a fast-to-slow fiber type transformation takes place in the diaphragm of patients with identical disease severity [14,23,88,123,124,125,126]. Such a phenotype renders the respiratory muscle more fatigue resistant, especially at the expense of the decreased ability to generate force shown by slow-twitch fibers [123,125]. Importantly, atrophy of fast-twitch fibers has also been recently reported in the peripheral muscles of severe COPD exhibiting nutritional abnormalities and significant muscle wasting [19]. In the diaphragms of COPD patients with a wide range of disease severity, atrophy of all fiber types was also described in previous studies [123,124,125,126,127]. Interestingly, reduced myosin heavy chain (MyHC) content and increased protein degradation via the ubiquitin-proteasome pathway have also been shown in the diaphragm [23,128,129,130] and vastus lateralis muscles [19,68] of COPD patients. The specific contribution of oxidative stress and protein carbonylation to skeletal muscle dysfunction in patients with COPD is reviewed below.

### 8.2. Biological Significance of Muscle Protein Carbonylation in COPD

In the last decade, it has been proposed that oxidative stress is a major contributor to muscle dysfunction in COPD patients, especially in those with a severe disease [14,15,16,17,18,19,20,21,22,23,24,25,26,27,28,29,30]. Several investigations have consistently demonstrated that under resting and exercise conditions, COPD patients exhibit higher levels of lipid peroxidation, oxidized glutathione, and protein carbonylation and nitration in their blood and both respiratory and limb muscles [14,15,16,17,18,19,20,21,22,23,24,25,26,27,28,29,30]. Chronic exposure to cigarette smoke also induced a significant rise in several oxidative stress markers including protein carbonylation in limb muscles of healthy smokers [17]. The sources potentially involved in the generation of ROS in muscles of patients with COPD have also been lately revealed. In this regard, mitochondria and membrane (NADPH oxidase) were shown to be the main molecular sources of ROS production in respiratory and limb muscles of severe COPD patients [19,23,27,131].

The development of oxidative stress has strong functional implications on the contractile performance of skeletal muscles and other clinical parameters of the affected patients. Systemic oxidative stress levels were directly related to quadriceps endurance time (a parameter of fatigue resistance) in severe COPD patients [20]. These authors also demonstrated that patients with hypoxemia exhibited greater levels of oxidative stress in their limb muscles, both at rest and after exercise, while showing a poorer quadriceps performance compared to healthy controls [22]. In another investigation [14], severe COPD patients developed greater levels of protein oxidation in their diaphragms, which inversely correlated with their respiratory muscle function and the degree of the airway obstruction. Several studies from our group [16,17,63] also showed that quadriceps muscle force was inversely related to the levels of protein oxidation being generated within those muscles. Importantly, body composition, which is a parameter of health status, and exercise capacity were also shown to be inversely related to protein oxidation levels within the vastus lateralis of patients with severe COPD [16].

The development of oxidative stress in skeletal muscles of COPD patients has long been postulated to be the result of enhanced inflammatory cell infiltration and cytokine production. Nevertheless, results obtained in our group [17,19,22,30,63,132,133] point towards the lack of a strong relationship between muscle oxidative stress and local inflammation among COPD patients. In fact, we reported that while local and systemic levels of inflammatory mediators are relatively low in patients with COPD regardless of their body composition, evidence of strong oxidative stress is consistently found in skeletal muscles and in the blood of these patients [17,19,22,30,63,133].

One possible mechanism whereby excessive ROS generation may adversely influence muscle contractile performance is via induction of posttranslational modifications that may result in reduced activity and increased proteolysis of key enzymes and proteins inside skeletal muscle fibers. As a matter of fact, it has been shown that the posttranslational oxidative modifications usually occur in critical amino acid residues of proteins sensitive to selective oxidation phenomena, which may result in loss of protein function [44,45,134,135,136,137,138], as well as in accelerated protein degradation by the proteasome [139,140]. Specifically, it has been consistently shown that structural proteins such as actin and myosin heavy chain (MyHC) [19,23], and enzymes such as creatine kinase and carbonic anhydrase-3 undergo severe oxidation within the respiratory and limb muscles of patients with COPD, both at rest and after exercise [15,16,17,19,23,141].

Structural proteins. Interestingly, the content of contractile MyHC was also decreased in muscles of COPD patients [19,23]. Despite the fact that a direct causal relationship cannot be established between enhanced MyHC carbonylation [23] and the reduced content of the protein [23,129,130] in respiratory muscles of severe COPD patients, it is likely that enhanced carbonylation may render MyHC protein more prone to be rapidly degraded by the proteolytic systems, which were shown to be significantly active in the diaphragms of those patients [129]. Furthermore, in another study [19], MyHC was also shown to be more carbonylated in the vastus lateralis of patients with severe COPD both with and without cachexia, while levels of the contractile protein were only significantly reduced in the latter patients. Actin structural protein has also been shown to be consistently carbonylated in muscles of severe COPD patients [16,17,19,23].

Enzymes. Importantly, protein and activity levels of enzymes involved in different aspects of muscle metabolism and adenosine triphosphate (ATP) preservation were significantly decreased in the patients compared to the controls [15,16,17,19,23,141]. Taken together, it would be possible to conclude that carbonylation of the protein side-chains may impair protein stability and function. However, the specific functional implications of enhanced oxidation and decreased creatine kinase activity to contractile performance of respiratory and limb muscles in COPD patients remain unclear, though are likely to play a relevant role. Actually, the absence of creatine kinase activity induced profound reductions in exercise performance [142] and myocardial dysfunction [143] in mice, leading to the conclusion that oxidative modifications of creatine kinase activity may also to muscle contractile dysfunction in COPD [17,19,23].

## 9. Protein Carbonylation in Cancer Cachexia Models

In advanced malignant diseases, cachexia defined as the loss of body weight, muscle atrophy, fatigue and weakness, and anorexia in the absence of a voluntary wish to lose weight, appears to be a common systemic manifestation, which always implies a poor prognosis [144]. Enhanced levels of oxidative stress were shown in muscles of several experimental models of cancer-induced cachexia [31,33,37,38]. Specifically, levels of markers of oxidative stress such as total reactive carbonyls and both HNE- and MDA-protein adducts, and protein tyrosine nitration were significantly greater in the gastrocnemius of cachectic rats bearing the Yoshida AH-130 ascites hepatoma, which is characterized by a rapid and progressive loss of body and muscle weights [31,33].

Uncoupling proteins (UCPs) are members of a family of mitochondrial carriers located in the inner mitochondrial membrane. Studies have clearly shown that UCP overexpression is associated with uncoupling of mitochondrial respiratory chain in isolated culture systems [32]. However, UCPs have also been shown to participate in several processes other than the energy mismatching characteristic of hypercatabolic states [145,146]. In line with this, UCPs were shown to counteract the damaging effects of ROS on tissues via a mechanism of HNE activation of UCP3 [147]. Additionally, in another study [32], UCP3 was shown to attenuate protein carbonylation rather than protein nitration in mouse myotubes exposed to two different models of ROS generation. However, the specific role of UCP3 on the oxidative stress-mediated muscle wasting process needs to be further elucidated in *in vivo* models of cachectic states.

## 10. Protein Oxidation in Cancer Cachectic Muscles

*Studies in humans.* In another recent study from our group (unpublished observations), patients with cachexia associated with two different respiratory conditions, lung cancer or COPD, also exhibited an increase in total protein carbonylation in the vastus lateralis and blood compartments compared to the healthy control subjects. Levels of superoxide anion and MDA-protein adducts were also greater in the blood and muscles, respectively, of both groups of patients than in the controls (unpublished observations). Again these results confirm the systemic nature of cancer cachexia, in which various types of body compartments were shown to be concomitantly affected in patients. Moreover, the size of type II fibers was also significantly reduced in the limb muscles of both groups of cachectic patients compared to the controls. Future research should identify the specific mechanisms whereby protein carbonylation induces muscle protein degradation, thus reducing fiber size, especially of fast-twitch fibers, and the cross-talk mechanisms, eventually involved in the systemic effects of cachexia associated with cancer and other chronic conditions in patients.

*Cachexia in COPD and lung cancer.* Potential differences in the phenotype and expression of oxidative stress markers in limb muscles and blood of patients with COPD- and lung cancer-induced have been recently explored in a study from our group [68]. Importantly, muscle (vastus lateralis) and systemic levels of reactive carbonyls and MDA-protein adducts were significantly and equally increased in patients with cachexia induced by either lung cancer or COPD [68]. Additionally, a significant rise in plasma levels of superoxide anion and muscle content of SOD isoforms 1 and 2, together with SOD activity was also detected in both groups of cachectic patients. These interesting observations led to the conclusion that the end-stages of the process of muscle wasting (cachexia) are characterized by a similar pattern of expression of oxidative stress markers in patients regardless of the initial triggers of each condition.

*Studies in animals*. Importantly, total protein carbonylation levels were shown to be increased in various muscles such as gastrocnemius, tibialis anterior, soleus, and heart of cachectic rats bearing the Yoshida ascites hepatoma [33]. Compared to control rodents, proteins involved in glycolysis, ATP production and distribution, carbon dioxide hydration, muscle contraction, and mitochondrial metabolism were more carbonylated in all limb muscles and heart of the cancer cachectic animals [33]. Furthermore, in the gastrocnemius of the tumor-bearing rats, the size of the fast-twitch muscle fibers was decreased and the immunohistochemical localization of carbonylated proteins was more prominent in these fibers compared to the slow-twitch [33]. The conclusions from this study [33] were that cancer cachexia alters redox balance in fast- and slow-twitch limb muscles and heart of rats, inducing oxidative modifications of key proteins involved in muscle structure and function. Moreover, experimental cancer cachexia also induced a reduction in the size of fast-twitch fibers in the gastrocnemius muscle, which seemed to have been associated with increased protein oxidation [33]. Future investigations should elucidate the precise mechanisms whereby enhanced carbonylation induces a reduction specifically in the size of the fast-twitch fibers. Indeed, in other conditions, a decrease in fast-twitch fibers was also shown to be a major characteristic structural feature in muscles [19,33,148,149,150,151]. In line with this, in rat skeletal muscles, the mitochondria of fast-twitch fibers were shown to produce higher amounts of superoxide anion than those of slow-twitch fibers [152]. Therefore, it is likely that greater amounts of ROS synthesized by type II fibers induce oxidative modifications to surrounding proteins that may render them more susceptible to degradation by cellular proteolytic mechanisms [46,153].

In line with the observations reported in limb muscles [19,31,33], various proteins involved in glycolysis, ATP production and distribution, carbon dioxide hydration, and muscle contraction were also shown to be modified by reactive carbonyls in the diaphragm of cachectic rats bearing the Yoshida ascites hepatoma (unpublished observations). As far as we are concerned, this would be the first investigation trying to describe the molecular events in the main respiratory muscle, the diaphragm, in an experimental model of cancer-induced cachexia. These results led to the conclusion that various types of muscles are similarly affected by oxidants in response to oncological cachexia.

## 11. Protein Carbonylation and Muscle Dysfunction in Sepsis

Sepsis is usually defined as the systemic response to serious infection, including several clinical manifestations such as fever, tachycardia, tachypnea, leukocytosis, and a localized site of infection. When hypotension or multiple organ failure occurs as a consequence of this syndrome the condition is called septic shock. Respiratory muscle failure is one of the most important causes of death in patients with sepsis and/or septic shock [154]. Moreover, diaphragmatic contractile dysfunction has also been demonstrated in animal studies of endotoxemia and septic shock models [34,35,36,40,154,155,156,157].

*Studies in animals.* Several mechanisms such as metabolic and hemodynamic factors, bacterial endotoxin, cytokines, arachidonic acid metabolism, nitric oxide, and oxidative stress were proposed to be involved in the sepsis-induced respiratory muscle dysfunction [154]. For instance, levels of total reactive carbonyls and HNE-protein adducts were shown to be greater in the diaphragms of endotoxemic rats compared to control muscles [34,40]. Furthermore, such markers of protein oxidation were further increased in the diaphragms of both endotoxemic and control rats as a result of the administration of chromium mesoporphyrin-IX, a potent inhibitor of heme oxygenase activity [34,40]. Additionally, heme oxygenase inhibition also led to a significantly greater reduction in diaphragmatic contractility in the endotoxemic rats than in the control animals [34,40]. These findings suggest that heme oxygenases are likely to play a significant role in the defense against protein oxidation and promotion of muscle contractile function in sepsis [34,40].

In another experimental model of endotoxemia, the potential beneficial effects of the antioxidant NAC on muscles were also evaluated [35]. In the study, endotoxemia was associated with a significant reduction in respiratory muscle force, as measured by maximal inspiratory pressure, while it increased levels of protein carbonylation and nitration in the diaphragm of the septic rats [35]. Interestingly, concomitant treatment with NAC resulted in a decrease in muscle protein carbonylation and nitration levels, an increase in Mn-superoxide dismutase protein content and activity, together with a substantial improvement in the respiratory muscle function of the septic rodents [35]. Taken together, it could be argued that protein carbonylation is likely to play a key role in the respiratory muscle contractile dysfunction elicited by sepsis or endotoxemia, at least in these animal experimental models [34,35,36]. Oxidation of key cellular molecules such as ryanodine receptors, creatine kinase and structural proteins (actin and myosin) are counted among the most relevant targets possibly involved in the sepsis-induced contractile dysfunction in experimental models [34,35,36]. The antioxidant NAC would improve muscle contractile performance through oxidant scavenging from key cellular structures in the myofibers of septic animals [35].

## 12. Carbonylated Proteins in Septic Muscles

*Studies in humans.* Recently, levels of protein oxidation and nitration have also been explored in the respiratory and limb muscles of patients with severe sepsis [39]. Importantly, protein carbonylation levels were significantly greater in the vastus lateralis of the septic patients compared to the controls, while levels of that marker did not differ between patients and controls in the respiratory muscles (external intercostals) [39]. Inflammatory events, indeed, took place in the muscles of the patients, while no significant differences were observed in muscle fiber type proportions or sizes in the analyzed muscles [39]. We concluded that the activity and type of muscles could account for the differences observed in the expression of oxidative stress markers seen in respiratory and limb muscles of patients with sepsis. Future research should identify the nature of the carbonylated proteins in muscles of patients with severe sepsis. Furthermore, studies attempting to assess the implications of carbonylation and oxidation on protein structure and function in muscles from septic patients are also warranted.

*Studies in animals.* The nature of the oxidatively modified proteins in the diaphragm muscle of endotoxemic rats has also been identified using proteomics analyses [36]. As such glycolytic enzymes such as aldolase A, glyceraldehyde 3-phosphate dehydrogenase, and enolase 3β, creatine kinase isoforms, carbonic anhydrase-3, contractile α-actin, and the mitochondrial ubiquinol-cytochrome c reductase were shown to be carbonylated in the diaphragm muscles of endotoxemic rats [36]. Importantly, the activity of the enzymes creatine kinase and aldolase were negatively correlated with the levels of carbonylation of the corresponding proteins within the rat diaphragms, suggesting that the oxidative modifications may alter their function, probably as a result of the oxidation of their active sites [36].

Interestingly, the nature of the HNE-protein adducts in the diaphragms of endotoxemic rats was also identified in another study [156]. Proteomics analyses revealed that enolase 3β, aldolase, triosephosphate isomerase-1, creatine kinase, carbonic anhydrase III, aconitase 2, dihydrolipoamide dehydrogenase, and electron transfer flavoprotein-ß were selectively modified by HNE in septic rat diaphragms [156]. In addition, *in vitro* exposure of enolase to HNE demonstrated a decrease in the enzyme activity in a dose-dependent fashion, implying that the oxidative modifications induced by HNE on this protein alter its function, which may have a major role in the sepsis-induced muscle dysfunction [156]. Taken together, these results suggest that carbonylation of key amino acids, especially those located in the active sites of the enzymes, may interfere with their activity. Identification of the specific sites of the oxidative modifications taking place in the muscle proteins of these models remains to be elucidated. Future research should focus on the identification of the specific active sites modified by oxidants in structural and functional proteins of septic muscles.

## 13. Conclusions and Future Perspectives

Oxidative stress, especially protein oxidation, induces modifications on key proteins involved in muscle contraction, metabolism and structure, which may alter the function of the target muscles. Several specific sites and proteins have been described as being more susceptible to suffer modifications by the action of oxidants. Importantly, oxidized proteins may also be more prone to being rapidly degraded by the proteolytic systems in skeletal muscle fibers. This is an important effect of protein oxidation on tissues, since it may contribute to enhancing protein breakdown in catabolic states such as in patients with advanced chronic conditions including cancer cachexia. Further research is needed in order to assess the extent of muscle function loss and mass that can be attributed to protein carbonylation in patients suffering from the conditions described in this review. Novel proteomics tools will help elucidate this question [57,109,158,159]. Additionally, identification of the exact amount of carbonylation required to induce loss of protein function and content should also be the focus of future research. The cellular signaling pathways that are more sensitive to the action of reactive carbonyls in muscles of the reported human conditions will also be a matter of research in future studies.

In view of the existing literature on the effects of oxidants on skeletal muscles, the use of antioxidant agents with potential clinical applicability will be of interest as shown in previous studies [35,62]. The analysis of the effects of antioxidant supplements on muscle performance in human conditions will require the design of rigorous experimental procedures in which sensitive markers of oxidative stress, outcome of clinical and physiological variables, and strict dietary control will have to be carefully selected [160]. Identification of the specific protein sites modified by ROS using well-validated redox proteomics approaches [158,161] in skeletal muscles will also be of relevance. Finally, identification of the impact of such alterations on tissue protein function and stability will also be important to the scientific community. In patients with chronic disorders, oxidative damage through oxidation and carbonylation of skeletal muscle proteins is a major etiological factor in the process of muscle wasting and dysfunction. Importantly, protein oxidation and muscle mass loss have also been consistently reproduced in experimental models of chronic conditions.

## Abbreviations

ATP: Adenosine triphosphate
COPD: Chronic obstructive pulmonary disease
DNA: Deoxyribonucleic acid
DNP: 2,4-dinitrophenylhydrazone
DNPH: 2,4-dinitrophenylhydrazine
O_2_^−·^: Superoxide anion
ELISA: Enzyme-linked immunosorbent assay
FEV_1_: Forced expiratory volume in one second
GSH/GSSG: Ratio of reduced to oxidized glutathione
HNE: Hydroxynonenal
H_2_O_2_: Hydrogen peroxide
HOO^·^: Hydroperoxyl radicals
iTRAQ: Isobaric tags for relative and absolute quantitation
MIP: Maximal inspiratory pressure
MDA: Malondialdehyde
NADPH: Nicotinamide adenine dinucleotide phosphate hydrogen
NAC: *N*-acetyl cysteine
NO: Nitric oxide
NOS: Nitric oxide synthase
NO_2_: Nitrogen dioxide
OH^∙^: Hydroxyl radicals
ROS: Reactive oxygen species
RNS: Reactive nitrogen species
SOD: Superoxide dismutase
UCP: Uncoupling protein
